# Treating binge eating and food addiction symptoms with low-carbohydrate Ketogenic diets: a case series

**DOI:** 10.1186/s40337-020-0278-7

**Published:** 2020-01-29

**Authors:** Matthew Carmen, Debra Lynn Safer, Laura R. Saslow, Tro Kalayjian, Ashley E. Mason, Eric C. Westman, Shebani Sethi

**Affiliations:** 1 The University of Michigan, Ann Arbor, MI USA; 2 Department of Psychiatry and Behavioral Sciences, Stanford University School of Medicine, 401 Quarry Road, Stanford, CA 94305 - 5723 USA; 3 Yale University School of Medicine, New Haven, CT USA; 4 The University of California San Francisco, San Francisco, CA USA; 5 Duke University School of Medicine, Durham, NC USA

**Keywords:** Binge eating, Food addiction symptoms, Obesity, Ketogenic diet, Dietary restriction

## Abstract

**Background:**

Many patients with obesity and comorbid binge eating symptoms present with the desire to lose weight. Although some studies suggest that dietary restriction can exacerbate binge eating, others show dietary restriction is associated with significant reductions in binge eating. The effect of a particular type of dieting on binge eating, the ketogenic diet (a high fat, moderate protein, very low carbohydrate diet), is not known.

**Case presentations:**

We report on the feasibility of a low-carbohydrate ketogenic diet initiated by three patients (age 54, 34, and 63) with obesity (average BMI 43.5 kg/m^2^) with comorbid binge eating and food addiction symptoms. All patients tolerated following the ketogenic diet (macronutrient proportion 10% carbohydrate, 30% protein, and 60% fat; at least 5040 kJ) for the prescribed period (e.g., 6–7 months) and none reported any major adverse effects. Patients reported significant reductions in binge eating episodes and food addiction symptoms including cravings and lack of control as measured by the Binge-Eating Scale, Yale Food Addiction Scale, or Yale-Brown Obsessive-Compulsive Scale modified for Binge Eating, depending on the case. Additionally, the patients lost a range of 10–24% of their body weight. Participants reported maintenance of treatment gains (with respect to weight, binge eating, and food addiction symptoms) to date of up to 9–17 months after initiation and continued adherence to diet.

**Conclusions:**

Although the absence of control cases precludes conclusions regarding the specific role of ketogenic diets versus other forms of dietary restriction, this is the first report to demonstrate the feasibility of prescribing a ketogenic diet for patients with obesity who report binge eating and food addiction symptoms. Further research should seek to reproduce the observed effects in controlled trials as well as to explore potential etiologies.

## Background

Dietary restriction in individuals with obesity who report binge eating and wish to lose weight is associated with mixed outcomes [1–3]. These inconsistencies are partly as a result of a lack of consensus regarding definitions of dietary restriction as well as diagnostic differences among study populations. For example, although dietary restriction may play a causal role in the development and maintenance of disordered eating in bulimia nervosa [4], studies specifically examining the effects of strict dieting in binge eating disorder (BED) have reported significant reductions and even remission of binge eating [5–8]. Re-introduction of normal dietary patterns is accompanied by an increase in binge eating, however the severity does not reach pre-treatment levels and many patients no longer meet criteria for BED [5, 7, 8]. According to the DSM-5, the key diagnostic features of BED include recurrent and persistent episodes of binge eating, marked distress regarding binge eating, absence of regular compensatory behaviors, and binge eating episodes associated with a variety of physical and psychological symptoms [9].

Often seen co-morbid with BED are food addiction symptoms of cravings and lack of control. Although food addiction is a controversial construct, the most widely used tool for assessing self-reported addictive symptoms in relation to food is the Yale Food Addiction Scale (YFAS)—which was developed by modeling the DSM-IV criteria for substance abuse [10]. Food addiction symptoms have been described as an addictive response to foods such as sweets and starches. These symptoms include much time spent obtaining food, feelings of withdrawal when off food, continued use despite knowledge of adverse consequences, important activities reduced or given up, repeated unsuccessful attempts to quit, and taken in larger quantities or longer periods than intended [11]. The prevalence of food addiction symptoms within individuals with obesity, using YFAS criteria, ranges from 15 to 20% [12–14] with rates up to 42% among candidates for bariatric surgery [15, 16]. While the extent of overlap between food addiction and BED is unclear, the subset of binge eaters with food addiction represent a more impaired group [17]. Higher levels of binge frequency have been associated with higher scores on the YFAS with severe food addiction identified as a score of at least a 6 [18]. However, there are currently no studies that estimate Food Addiction prevalence or incidence separate of the obese condition. There are also no studies validating consistent food addiction diagnostic criteria, and how these should differ from Binge Eating Disorder diagnostic criteria. The impact of dietary restriction among patients who report greater food addiction symptoms has not been clearly established, however a study has shown in a general population an increase in food cravings in restrained eaters compared to unrestrained eaters of specific foods, such as chocolate [19].

Ketogenic diets are high-fat, low-carbohydrate, moderate-protein dietary patterns in which the body’s principal energy source is fat. This falls into an approximate range of 60% fat, 10% carbohydrates, and 30% protein. Typical energy intake per day on a ketogenic diet is at least 5040 kJ. If individuals adhere to this dietary pattern, nutritional ketosis results, which can be defined by sustaining blood beta-hydroxybuterate levels between .5 mmol/L and 3 mmol/L [20]. Sustained nutritional ketosis induces a series of physiological changes involving appetite suppression, lower hunger, greater satiety, greater rates of lipolysis, reduction of lipogenesis and increased metabolic costs of gluconeogenesis and thermic effect of proteins [21]. Studies demonstrating ketone-induced appetite suppression and enhanced satiety provide a theoretical basis for prescribing a ketogenic diet for individuals with obesity who report binge eating and food addiction symptoms [22]. Such patients often endorse an inability to withstand increased levels of hunger, disturbances of satiety (e.g., “never feeling full”), food cravings, and urges to binge eat, and cite these experiences as preventing them from achieving weight loss.

Hence, we report the findings of prescribing a ketogenic diet to three patients with obesity who endorsed binge eating, food addiction symptoms upon symptom presentation and reported a desire to lose weight and improve metabolic health. Selection of these patients by the authors was not based on amount of weight lost, but on endorsement of specific initial symptom presentation of binge eating or food addiction symptoms and co-morbid with the disease of obesity. Thus, these are the 3 cases reported. It was thought by the clinicians that the patients were suitable to try this approach given the treatment resistance and metabolic abnormalities the patient faced.

After describing the cases and their associated outcomes, we briefly reviewed the literature to explore possible biological mechanisms that may explain these outcomes. See Table 1 for demographic data of each patient.

**Table 1:** Demographic data

Case	Age	Gender	Race	Education
1	54	F	African American	College
2	34	M	Caucasian	College
3	62	F	Caucasian	College

## Case presentations: case 1

### Baseline interview

A 54-year-old, African American, college-educated postmenopausal female presented with obesity and self-reported binge eating and food addiction symptoms. She reported a history of sporadic attempts to lose weight with exercise, using over-the-counter weight loss pills, and attempting diet programs such as Weight Watchers. She reported that despite these efforts, her binge eating had only worsened. Her binge frequency was 1–3 times daily with estimated 14 + times per week consistently over years. She believes that this contributed to her recurrent depression and history of suicide attempts.

She denied any current use of illicit substances but did report a history of addiction to cocaine and nicotine during her thirties, which she overcame with the help of Narcotics Anonymous. Other notable history included excoriation disorder (chronic skin-picking). Past treatment included bupropion and cognitive behavioral therapy (CBT) for clinical depression, which put her in remission for several years. She did not endorse current suicidal ideation or a history of sexual trauma or physical abuse. She reported family history of illicit substance use, obesity, type 2 diabetes mellitus, Alzheimer’s disease, hyperlipidemia, and hypertension.

### Baseline testing

See Table 2 for baseline weight and BMI. Her depression score on the Patient Health Questionnaire (PHQ-9) was 20 out of 27, indicating severe depression. She also had severe binge eating symptoms meeting DSM-5 criteria for binge eating disorder (BED), with a Binge-Eating Scale (BE) score of 35 out of 46 [23, 24]. She reported severe obsessive-compulsive symptoms regarding food, with a Yale-Brown Obsessive-Compulsive Scale modified for Binge Eating (YBOC-BE) score of 39 out of 40 [25]. She reported severe food addiction symptoms with a Yale Food Addiction Scale (YFAS) score of 10 out of 12 [10].

**Table 2:** The effects across time of a ketogenic diet on binge eating, depression, food addiction symptoms and weight

Variable	Cases	Baseline	Change after 6–7 months	Change after 9–13 months	Change after 17 months
Patient Health Questionnaire-9 (depression subscale score)	Case 1	20	−19		
	Case 2	–	–		
	Case 3	–	–		
Binge-Eating Scale	Case 1	35	− 31		
	Case 2	–	–		
	Case 3	–	–		
Yale-Brown Obsessive-Compulsive Scale (modified for Binge Eating)	Case 1	39	− 37		
	Case 2	–	–		
	Case 3	–	–		
Reported Binge Eating Episodes and Frequency	Case 1	1–3 times daily; 14 times per week	−14 weekly	−14 weekly	−14 weekly
	Case 2	1–2 times daily; 8–11 times per week	− 8 to − 11 weekly	− 8 to − 11 weekly	−8 to − 11 weekly
	Case 3	1–2 times daily; 8–10 times per week	− 8 to − 10 weekly	−7 to – 9 weekly	−8 to − 11 weekly
Yale Food Addiction Scale	Case 1	10	− 9		
	Case 2	7	−6		
	Case 3	6		−4	
Weight (kg)	Case 1	115.7	−17.0	− 26.3	− 28.2
	Case 2	145.2	−20.4	−19.1	
	Case 3	110.2	−10.0	−10.9	
Weight (% weight change)	Case 1		−14.7	−22.7	−24.4
	Case 2		−14.0	−13.2	
	Case 3		−9.1	−9.9	
BMI (kg/m^2^)	Case 1	43.1	−4.6	−9.1	−10.0
	Case 2	47.1	−6.8	−6.0	
	Case 3	40.4	−3.8	−4.0	

### Prescribed protocol

Patient was seen by Dr. Shebani Sethi (a psychiatrist and obesity medicine physician) and by Dr. Eric Westman in his outpatient Duke Lifestyle Medicine Clinic (an internist and obesity medicine physician). The patient was carefully taught to follow a ketogenic diet, with carbohydrates as the only restriction to 20 g a day. We instructed the patient to eat whole foods, not processed, including meat and eggs, 4 oz of hard cheese, 2 cups of assorted salad vegetables, and 1 cup of non-starchy low-carbohydrate vegetables per day. A list of vegetables, salad, and protein food options were provided. We instructed the patient to not count calories and to eat these foods until full then stop. We informed the patient that during the first few weeks (keto-adaptation phase) she might experience side effects including headache, fatigue, constipation and other symptoms associated with weight loss as her body adapted from metabolizing carbohydrates to metabolizing fats (ketosis). Binge eating was measured by clinical interview and the YFAS, YBOC-BE/BES scales were completed in clinic by paper and pen. Vitals were taken by nursing staff. Patient was seen regularly at follow-up visits every 2 weeks and asked to complete dietary recall questionnaires and track the number of carbohydrates per day. She reported adhering to an average of 20-30 g per day. After 2 months, her interval for follow up was monthly, and after month 6, was every 3–6 months as needed.

### Patient outcome

After 6 months of following the ketogenic diet without any major adverse effects (she did endorse keto adaptation symptoms of temporary headache and fatigue in the first week, which then resolved In the second week), the patient reported that she was no longer binge eating (BES score reduced to 4 of 46). She experienced several other improvements as well, including a cessation of excoriation symptoms (picking and scratching at skin and fingernails), a feeling of empowerment, and greater control of her eating behaviors. She reported continued obsessive thoughts about food, but she felt more able to resist them (YBOC-BE score reduced to 2 of 40; YFAS score reduced to 1 of 12). In addition, she evidenced improvement in psychological functioning, including a reduction in her depressive symptoms (PHQ-9 score reduced to 1 of 27). After 6 months on the ketogenic diet, she lost 18.1 kg, resulting in a 7.7 kg/m^2^ reduction in BMI from 43.9 kg/m^2^ (morbid obesity, class 3) to 36.2 kg/m^2^ (obesity, class 2). At her last follow-up appointment (12 months from the initiation of the diet) she reported that she was continuing to follow the diet and had maintained her weight loss. She reported not having difficulties with adhering to the diet.

## Case presentations: case 2

### Baseline interview

A 34-year-old, Caucasian, college-educated male presented with obesity and self-reported history of binge eating disorder and food addiction symptoms. The patient described a lifelong history of obesity that progressively worsened throughout his teenage years and young adulthood. He also developed hyperlipidemia and obstructive sleep apnea. He described a complete lack of control over his eating, including eating in the absence of hunger and significant guilt after binge episodes. Reported binge frequency was 1–2 times per day with estimated 8–11 times per week. In particular, he ate pizza frequently until it was painful for him. He suffered from reflux and would on occasion vomit (not in a compensatory fashion) due to physical overconsumption. He denied the use of illicit substances and did not smoke. In addition, he reported infrequent alcohol use. His family medical history was unknown.

### Baseline testing

See Table 2 for his baseline weight, BMI, and Yale Food Addiction Scale score. No other self-report measures or scales were administered.

### Prescribed protocol

The patient was seen by Dr. Tro Kalayjian, an internist and obesity medicine physician in his clinic at Yale. The patient indicated that he wanted to try a ketogenic diet for weight loss. Self-reported binge eating was present which met DSM-5 criteria for BED, but was seen in a medicine clinic, thus binge eating was not the focus of the behavioral intervention. Patient was instructed to follow a ketogenic diet similarly described in introduction. Carbohydrates were restricted to under 30 g per day but protein or fat was not limited. Patient was not asked to count calories. The patient had no other food restrictions. The patient was educated on food quality and hyper-palatability of processed foods and was encouraged to eat unprocessed food. The patient was also encouraged to make ketogenic versions of the foods he enjoyed, for example including pizza crust made from almond flour, protein bars in lieu of processed and refined sugar cookies, and protein chips instead of potato chips. Patient was instructed to include whole foods, fish, eggs, chicken, seafood, low carbohydrate fruits, and a list of non-starchy vegetables and salad. Patient was informed of temporary keto-adaptation side effects as described above in case 1. Vitals were measured by nursing staff and patient was seen weekly for 16 weeks then as patient directed for follow-up.

### Patient outcome

After following the ketogenic diet for 6 months with no reported major adverse effects (except for temporary fatigue in the first week), the patient’s YFAS score was 1; his food addiction symptoms were almost completely resolved. He reported no binge eating episodes and also described a lack of food cravings since initiating the treatment. His reflux symptoms resolved after the first month. After 6 months on the ketogenic diet, he lost 20.4 kg, resulting in a reduction in BMI from 47.1 kg/m^2^ (morbid obesity, class 3) to 40.3 kg/m^2^ (also morbid obesity, class 3, but close to the BMI cut-off of less than 40 kg/m^2^ for class 2 obesity). Thirteen months after initiation of the diet, he reported sustained adherence, with maintained improvements in binge eating and food addiction symptoms. Adherence was measured regularly by dietary recall, tracking of carbohydrates, and by blood ketone measurements showing numbers between 0.5–5.0, indicating nutritional ketosis.

## Case presentations: case 3

### Baseline interview

A 62-year-old, Caucasian, college-educated female with obesity and self-reported history of binge eating disorder and food addiction symptoms. She also developed hyperlipidemia, hypertension, and a self-diagnosed history of depression. She denied a history of illicit substance use, including nicotine or alcohol. She reported a history of childhood physical and sexual abuse by a step-mother for which she had sought counseling. She reported a lifelong issue controlling her chocolate intake. Reported binge frequency was 1–2 times per day with estimated 8–10 times per week. Her family medical history included alcohol abuse, obesity, hypertension, and type 2 diabetes.

### Baseline testing

See Table 2 for her baseline weight, BMI, and Yale Food Addiction Scale score. No other self-report measures or scales were administered. She did meet DSM-5 criteria for binge eating disorder.

### Prescribed protocol

The patient was seen by Dr. Tro Kalayjian. The patient indicated that she wanted to try a ketogenic diet. A ketogenic dietary intervention similar to case 2 was taught. Patient was instructed to include whole foods, fish, eggs, chicken, seafood, low carbohydrate fruits, and a list of non-starchy vegetables and salad. Patient was informed of keto-adaptation side effects as described in case 1. Vitals were measured by nursing staff and patient was seen weekly for 16 weeks then as patient directed for follow-up.

At first, a time-restricted eating window of 6 h was also suggested as a supplement to the dietary intervention, which the patient did not implement until week 6. It was indicated that she could implement the time-restricted eating window whenever she felt ready, if she even desired to at all. It was encouraged to her to maintain a box of very low-carbohydrate chocolate protein bars in the home and to eat them without restriction. When her food addiction symptoms of cravings and lack of control improved after several months on the ketogenic dietary intervention, the patient then supplemented with a time restricted feeding schedule where food intake was restricted to 6 h of the day.

### Patient outcome

After following the ketogenic diet for 7 months, she reported no difficulties adhering to the eating protocol and no major adverse events. She initially experienced some headache and fatigue during the first week, which resolved in the second week. She lost 10 kg over this time period, resulting in a 3.8 kg/m^2^ reduction in BMI from 40.4 kg/m^2^ (morbid obesity, class 3) to 36.6 kg/m^2^ (obesity, class 2). After following the ketogenic diet for 9 months, her YFAS score reduced to 2 indicating a reduction from severe to mild food addiction symptoms. She no longer experienced guilt and felt completely in control of her eating behavior; we observed only 1 binge episode over 9 months. She reported following the ketogenic diet at 6 months after treatment with a total weight loss of 10.9 kg (13 months after diet initiation). Her BMI and weight at 6 months follow-up were 36.4 kg/m^2^ and 99.3 kg, respectively. She stated she frequently eats only one meal a day without experiencing any significant hunger, nor feelings of deprivation, or desire for chocolate. Adherence was measured regularly by dietary recall, tracking of carbohydrates, and by blood ketone measurements showing numbers between 0.5–5.0, indicating nutritional ketosis.

## Discussion and conclusions

The results of this small case series support the potential feasibility of using a low carbohydrate ketogenic diet for patients presenting with obesity and self-reported binge eating and food addiction symptoms. All patients were able to adhere to the ketogenic diet with no reported major adverse side effects. Patients reported significant reductions in self-reported symptoms of binge eating episodes and food addiction (as measured by YFAS, Y-BOC-BE, or reported cravings). Additionally, the patients lost 10–24% of their body weight. Participants reported maintenance of treatment gains to date of over 9–17 months after initiation of the diet. In addition, when accompanied by co-morbid depressive symptoms, substantial improvements in mood symptoms corresponded with a decline in the PHQ-9.

Ketogenic diets have long been used as an effective treatment for pediatric epilepsy [26]. Randomized clinical trials have shown that a less stringent, low-carbohydrate ketogenic diet, such as the intervention used for these patients, can be effective in treating obesity and reversing type 2 diabetes [27–29]. Ketogenic diets have also been reported to be helpful in other clinical conditions, with case series documenting improvement or resolution of gastro-esophageal reflux disease, irritable bowel syndrome, and Crohn’s disease [21, 30–32]. Less is known regarding the effect of ketogenic diets on mental health. Some have observed an improvement in mood in bipolar patients maintained on long-term ketogenic diets, hypothesizing that diet-induced nutritional ketosis mimics the action of mood stabilizers in reducing intracellular sodium and calcium [33]. Others have documented improvement in symptoms of psychosis in patients with schizophrenia [34, 35].

The mechanisms by which a low-carbohydrate ketogenic diet influence binge eating and food addiction symptoms are complex, multifactorial, and may involve the effects of nutritional ketosis and its ensuing metabolic effects. Literature review suggests potential mechanisms may involve changes in hormone systems which work to increase satiety, improve leptin sensitivity, and reduce appetite. This may include higher circulating levels of CCK, PYY, and decreases in ghrelin and leptin [36–44]. Other potential mechanisms the literature suggests include changes in metabolism of excitatory amino acids leading to gamma-Aminobutyric acid (GABA) inhibition and brain-derived neurotrophic factor (BDNF) expression [45]. A complete discussion of the literature review on potential mechanisms are beyond the scope of this case series paper.

Limitations of this case series should be noted. Without a control condition, it is not possible to distinguish between the effects of dietary restriction generally, versus a low-carbohydrate ketogenic diet specifically, in driving reductions in weight and binge eating symptoms. Nor can we determine, without larger samples, whether the diet’s high acceptability among these three patients is typical. Blood ketones were not measured in case 1, but patients were required to complete dietary recalls at follow-ups and report tracking of their total carbohydrates per day. With nutritional ketosis, appetite suppression and satiety are expected effects, thus these symptoms were routinely monitored and obesity medicine physicians clinically distinguish between those in or out of ketosis based on the expected symptomology of ketosis. It is possible that having severe binge eating and food addiction symptoms, which placed these patients into a more impaired subgroup of patients, led them to experience greater benefits than those who were less impaired would have experienced. The relief of experiencing abstinence from cravings may have felt like a worthwhile tradeoff for this group, unlike less symptomatic patients. Additionally, although the Binge Eating Scale (BES) scale has been validated to assess presence of binge eating symptoms, there is no research to show that it can validly diagnose cases of Binge Eating Disorder like the DSM-5 [24]. Finally, as patients were treated by different providers, only the YFAS was uniformly administered to all.

Despite these limitations, we feel this case series demonstrating the feasibility of low carbohydrate ketogenic diets among three individuals with obesity and self-reported binge eating and food addiction symptoms is important, given the lack of prior studies and the importance of understanding the impact of this specific diet for patients with such highly disordered eating.

In summary, research on the role of dietary restriction in the treatment of obesity with co-morbid disordered eating has been limited. We propose that achieving nutritional ketosis through macronutrient dietary restriction of carbohydrates in the treatment of obesity and self-reported binge eating and food addiction symptoms is potentially feasible. Patients experienced reductions in binge eating and food addiction while also losing weight. The findings are limited by the lack of a control group. Although the mechanisms by which sustained ketosis affect appetite and satiety are not definitively understood, they may include hormone-mediated impacts. Clinicians may wish to consider a low-carbohydrate, ketogenic diet for patients with obesity who report binge eating and food addiction symptoms, especially when other interventions have failed. Further research should seek to reproduce the observed effects in controlled trials as well as potential etiologies.

## Data Availability

Data sharing is not applicable to this article as no datasets were generated or analyzed during the current study.

## Abbreviations

BDNF: Brain-derived neurotrophic factor
BED: Binge eating disorder
BES: Binge-eating scale
BMI: Body mass index
CBT: Cognitive behavioral therapy
CCK: Cholecystokinin
GABA: Gamma-Aminobutyric acid
PHQ-9: Patient Health Questionnaire
PYY: Polypeptide Y
YBOC-BE: Yale-Brown Obsessive Compulsive Scale modified for Binge Eating
YFAS: Yale Food Addiction Scale
