# Correction: Treating binge eating and food addiction symptoms with low-carbohydrate Ketogenic diets: a case series

**DOI:** 10.1186/s40337-023-00881-1

**Published:** 2023-09-29

**Authors:** Matthew Carmen, Debra Lynn Safer, Laura R. Saslow, Tro Kalayjian, Ashley E. Mason, Eric C. Westman, Shebani Sethi

**Affiliations:** 1 The University of Michigan, Ann Arbor, MI USA; 2 Department of Psychiatry and Behavioral Sciences, Stanford University School of Medicine, 401 Quarry Road, Stanford, CA 94305 - 5723 USA; 3 Yale University School of Medicine, New Haven, CT USA; 4 The University of California San Francisco, San Francisco, CA USA; 5 Duke University School of Medicine, Durham, NC USA

**Correction to: Journal of Eating Disorders (2020) 8:2** 10.1186/s40337-020-0278-7

Following the publication of the original article [1] the corresponding author requested the modification of her name from "Shebani Sethi Dalai" to "Shebani Sethi".

The correct author name appears in the author list of this Correction. The original article has also been corrected.
